# Solving Community SARS-CoV-2 Testing With Telehealth: Development and Implementation for Screening, Evaluation and Testing

**DOI:** 10.2196/20419

**Published:** 2020-10-16

**Authors:** Aditi U Joshi, Resa E Lewiss, Maria Aini, Bracken Babula, Patricia C Henwood

**Affiliations:** 1 Thomas Jefferson University Hospital Philadelphia, PA United States

**Keywords:** telehealth, telemedicine, disaster planning, pandemic, COVID-19, SARS-CoV-2, emergency medicine, testing

## Abstract

**Background:**

Telehealth has emerged as a crucial component of the SARS-CoV-2 pandemic emergency response. Simply stated, telehealth is a tool to provide health care from a distance. Jefferson Health has leveraged its acute care telehealth platform to screen, order testing, and manage patients with COVID-19–related concerns.

**Objective:**

This study aims to describe the expansion and results of using a telehealth program to increase access to care while minimizing additional potential exposures during the early period of the COVID-19 pandemic.

**Methods:**

Screening algorithms for patients with SARS-CoV-2–related complaints were created, and 150 new clinicians were trained within 72 hours to address increased patient demand. Simultaneously, Jefferson Health created mobile testing sites throughout eastern Pennsylvania and the southern New Jersey region. Visit volume, the number of SARS-CoV-2 tests ordered, and the number of positive tests were evaluated, and the volume was compared with preceding time periods.

**Results:**

From March 8, 2020, to April 11, 2020, 4663 patients were screened using telehealth, representing a surge in visit volume. There were 1521 patients sent to mobile testing sites, and they received a telephone call from a centralized call center for results. Of the patients who were tested, nearly 20% (n=301) had a positive result.

**Conclusions:**

Our model demonstrates how using telehealth for a referral to central testing sites can increase access to community-based care, decrease clinician exposure, and minimize the demand for personal protective equipment. The scaling of this innovation may allow health care systems to focus on preparing for and delivering hospital-based care needs.

## Introduction

The SARS-CoV-2 (COVID-19) pandemic presented the need to rapidly evolve the traditional in-person care model to distance treatment. Due to infection control considerations for clinicians and patients in higher-risk health care settings as well as worldwide shortages of personal protective equipment (PPE), telehealth became a crucial component to emergency health care [1-3]. Telehealth involves a remote medical encounter between two clinicians or between a patient and a clinician, and can be used for direct patient care at home, remote consults to specialists, and chat visits asynchronously [4-9]. Due to the current pandemic, telehealth has been quickly leveraged to safely keep people at home, disseminate information, allow decisions around testing to be made, coordinate testing when appropriate, and risk stratify patients for evidence-based and resource efficient care. In the time of COVID-19, the benefit of telehealth seems obvious: a patient can be seen remotely, assessed, and evaluated for need of testing, all without a visit to the doctor’s office or to the emergency department (ED). A remote visit potentially prevents an in-person patient care visit and facilitates timely and responsive patient-centered care [10-12].

Jefferson Health, based in Philadelphia, Pennsylvania, created a system-wide program, JeffConnect, in 2015 that included an on-demand program, remote consults, scheduled visits for postoperative or primary care, and virtual rounds. When cases of SARS-CoV-2 abruptly increased around the world, Jefferson Health prepared. The health care system’s organizing plan was similar to that of other hospital settings: source more PPE; create further surge capacity; procure surge ventilators, critical equipment, and medications; streamline evaluation and management protocols; and cross-train staff. Essential to this plan was the additional incorporation of the on-demand acute care telehealth program, hereby referred to as JeffConnect, staffed by emergency medicine (EM) physicians already licensed in Pennsylvania, New Jersey, and Delaware. The institution was uniquely positioned to rapidly leverage this as an access point for care and link to SARS-CoV-2 testing. Although national telehealth companies also offer expanded services, they generally do not have local community integration and are often unable to offer testing and follow-up that is geographically linked to patient location. Jefferson Health, with its JeffConnect program and expanded services within its geographic region, did not have this limitation.

The first COVID-19 cases in Pennsylvania were reported on March 6, 2020. A state of emergency was declared in New Jersey on March 9, 2020, and a public outreach campaign regarding the ability to use telehealth for screening and an emphasis on the need for people to socially distance was launched at the same time. Jefferson Health (located in southeastern Pennsylvania and southern New Jersey) saw the increase in telehealth patient calls starting simultaneously early March.

Herein we aim to outline our real-time telehealth intervention at the start of the COVID-19 case surge in the region. We delineate how to integrate telehealth and testing sites to decrease clinician and patient exposure in a health care setting, maximize efficient use of PPE, and increase safe access to COVID-19–related testing and management in the community setting.

## Methods

### Program Overview

With the intention of preventing unnecessary COVID-19 exposures, many primary care providers, onsite clinics, and urgent cares in the region began to direct patients to consider telehealth first for noncritical complaints. JeffConnect is staffed 24 hours a day by physicians and sees a variety of acute care complaints, with visit times averaging less than 10 minutes. Patients require an internet connection and a smartphone, tablet, or computer, or access to one to use the platform. They pay the one-time out-of-pocket fee, or a reduced copay if they are a Jefferson employee. Due to this convenience, patients were directed to JeffConnect as the first viable solution for COVID-19 complaint screening and evaluation.

At the same time, a team composed of infectious disease specialists, health system clinical leadership, and occupational medicine leadership created screening protocols to determine who should be tested from the community, which groups of employees should be tested if they first contacted JeffConnect, and who could stay at home with guidance on self-isolation (due to limited testing capacity). Initial screening protocol developed during implementation considered limited testing capacity and prioritized COVID-19 infections in patients who presented with fever or symptoms of acute respiratory illness, were at the highest risk of exposure, were at a higher risk of developing severe disease, or were at a high risk of exposing others. Testing was recommended for patients who were symptomatic with one of the following: close contact with confirmed case, international travel within 14 days, at risk for severe disease including chronic medical conditions, residents of congregate settings, health care workers, patients who were hospitalized, and those presenting with acute respiratory illness without apparent cause. During the study period, patients who were asymptomatic were not recommended for testing.

Once mobile testing sites were operationalized and linked to JeffConnect on March 13, 2020, a SARS-CoV-2 lab order was placed in the electronic health record order entry system. After the order was placed by a clinician, the JeffConnect visit ended. Patients were instructed to contact a centralized call center, named Seamless Access. Seamless Access was a hub for patients to coordinate their on-site testing. The call center group completed the patient registration, confirmed that an order was placed, provided directions and times to present to a testing site, and provided patient education on what to expect upon arrival and in the follow-up. 

All test results from the electronic health record were routed to a dedicated results follow-up team and separated into positive and negative result pools. Data was extracted from the electronic medical record and stored on secure systems consistent with medical center policies. Patient data was deidentified for aggregate analysis. Every patient tested was contacted via telephone and received counseling on results, a re-evaluation of symptoms, and advice on when to return to work or seek additional care. For positive results, patients were contacted by a designated primary care physician or nurse practitioner. For negative results, patients were contacted by primary care nurse care coordinators. Home isolation for patients who were symptomatic was emphasized regardless of the result, given the possibility for false-negative results. Patients with access to the online patient portal were able to view their results immediately and received additional written instructions based on a positive or negative result. Each week, the number of patients, number of SARS-CoV-2 tests performed, and positive results were tracked for quality improvement processes and to inform considerations on testing capacity.

### Staffing Telehealth

**Figure 1:**
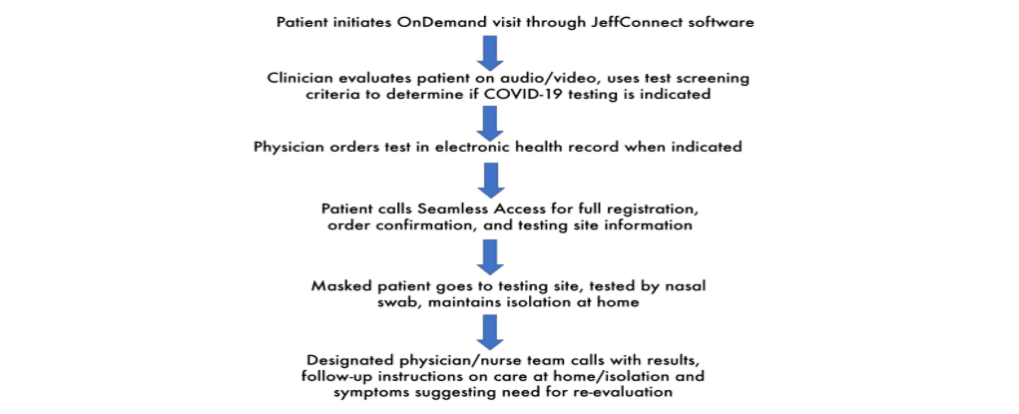
JeffConnect screening and testing process.

Considering more limited access to ambulatory visits, patients rapidly sought care via JeffConnect for COVID-19–related complaints as well as for other acute complaints that they were not able to see their primary care physician about. The goal was for patients who were generally well to be evaluated and determine if they required testing or were safe to stay home (Figure 1).

Due to the opening of the testing sites and the aforementioned goals, the prior staffing model of one EM physician managing JeffConnect visits, even with the expectation of seeing four patients an hour, was not sufficient to keep up with the abrupt increase in patient volume. JeffConnect’s leadership had to scale their process quickly to remotely evaluate a large volume of patients. To allow patients to be seen in a timely manner, during the weekend of March 13-15, 2020, 150 new physicians from internal medicine (IM), family medicine (FM), anesthesia, and EM advanced practice providers (APPs) were trained remotely using Zoom (Zoom Video Communications, Inc) and myJeffHub, an internal video education platform accessible to all employees. New clinician accounts were created by the JeffConnect Medical Director and all clinicians were given the training videos, tip sheets, screening protocols, and Jefferson policies.

The IM and FM physicians added additional coverage from the hours of 7 AM to 10 PM starting on March 15, 2020. IM and FM physicians, due to licensing constraints, only covered patients in Pennsylvania, while the EM staff would cover Pennsylvania, New Jersey, and Delaware. Other clinicians (anesthesia and APPs) were kept in reserve as needed for surge capacity. For EM faculty, contingency planning included ensuring those in high-risk groups such as those 60 years and older or who were immunocompromised were also trained for JeffConnect to convert in-person shifts to telehealth. The IM and FM practices converted a number of their patient visits to telehealth or rescheduled routine visits for later. At that time, there was no emergency state reciprocity for licensure, so most of the additional physicians covered patients from Pennsylvania only. The newly trained clinicians and the existing telehealth EM physician were able to meet the increased patient demand. 

Over the next week, the ED redistributed staff physicians and APPs to account for the fluctuating patient volumes. Clinicians deemed high risk for COVID-19 complications were restaffed to telehealth. JeffConnect leaders added a swing shift to increase the IM and FM staffing from 10 AM to 7 PM. New Jersey amended its licensing process, allowing for emergency temporary licensure. This allowed more physicians to provide telehealth to New Jersey patient callers. 

During the testing period, we evaluated the visit volume, number of SARS-CoV-2 tests ordered, and number of positive tests. JeffConnect visit volumes from the same period in 2019 and the 5-week time interval preceding this 5-week study period were evaluated for comparison. Descriptive statistics were used in the analysis.

## Results

**Figure 2:**
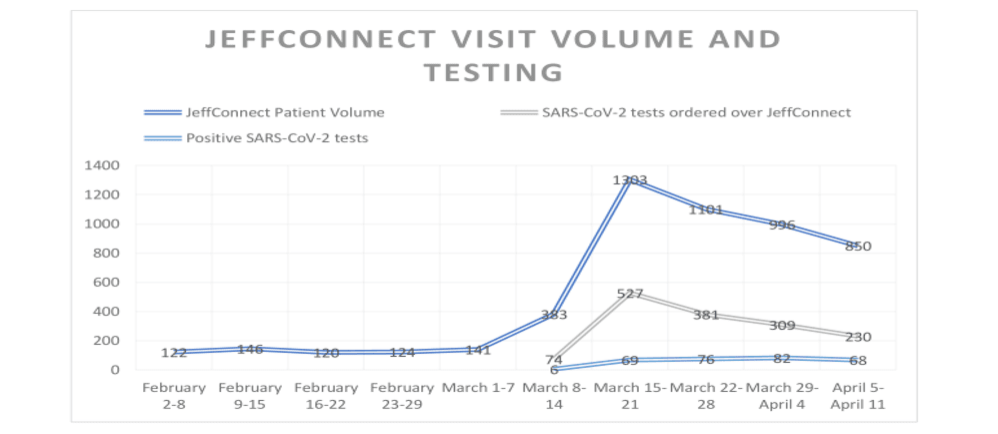
JeffConnect visit volumes and testing.

In the telehealth screening and testing study period (March 8, 2020, to April 11, 2020), 4663 patients were evaluated and 1521 (33%) were recommended for SARS-CoV-2 testing. Peak volumes for visits and testing occurred the week of March 15-21 with 1303 visits and 40% (n=527) of those patients sent for testing (Figure 2).

There was an increase in telehealth patient volume from 321 visits from March 8 to April 11 in 2019 to 4663 visits during the same dates in 2020. There were 653 total visits in the 5 weeks preceding the study period (February 2 to March 7, 2020), compared to 4663 during the study period. This represents a greater than seven-fold increase in visit volume. Of the 1521 patients that were tested, nearly 20% (n=301) had a positive result.

## Discussion

### Principal Findings

The abrupt rise in telehealth visit volume and COVID-19–related testing need, as well as the approximately 20% (301/1521) positive rate during our study period mirrors the numbers in Pennsylvania, with a 18% positive rate in the state during the same time period (129,207 SARS-CoV-2 tests with 23,340 positive cases in Pennsylvania) [13]. This coincides with the initial surge of COVID-19 cases in the northeast of the United States during this time period [14]. This suggests our screening algorithms and indications for testing, considering constrained testing resources, were similar to those being used in the region during the study period [13].

It is unclear how many in-person evaluations were prevented, and many of the new patients to JeffConnect were seen for evaluation of COVID-19 symptoms or exposures. We believe our surge in volume was also due to patients seeking care for other urgent complaints via telehealth to avoid in-person contact with the health care clinicians. The goal of quickly scaling telehealth capacity was to keep viral exposure at a minimum, and we suspect that receiving at-home screening kept patients from exposing others in person and potentially spared exposure for concerns not related to COVID-19. Considering the ongoing nature of the COVID-19 pandemic, it is not yet clear whether our region has reached its ultimate peak surge, and time will tell if this intervention significantly impacts access to care, testing, and appropriate isolation. 

### Process Replication

The JeffConnect telehealth COVID-19 innovation came together by the alignment of multiple factors. The region had more time than some other regions: time to write, organize, and execute health system–wide policies, and to evolve previous emergency management preparations for this specific context. As already stated, Jefferson had a pre-existing telehealth structure including and not limited to a platform, a workflow process, training guides, courses, and a quality assurance process. This allowed the efficient and rapid scale-up to meet the need for more clinicians to accommodate the increased number of patient visits. We were able to use existing staffing and electronic health record infrastructure to create multiple outpatient testing sites near these hospitals and urgent cares.

The commitment to resourcing this service to scale rapidly led to a successfully evolved program within just a few days. None of the challenges have been insurmountable. To date, the telehealth clinicians’ group can see patients in an efficient and timely manner, and direct them for immediate testing at one of seven sites. For systems seeking to replicate this process, we recommend the following:

- Establish patient care and testing algorithms that clearly identify who needs to be tested, considering local testing resources
- Implement a telehealth software based on a clearly identified use case. Due to the pandemic, many companies have created quick and discounted implementations, and clear definition of need will determine which solution is the right one.
- Train clinicians in telehealth: on technology, screening processes, and converting in-person encounters to virtual care
- Identify physical sites that can be used for walk-up or drive-thru testing, which are close to existing health system infrastructure and convenient for patients to access while maintaining infection prevention practices
- Identify and train staff at the testing sites for clear and specific roles
- Identify the appropriate testing equipment and keep count of testing swabs being used relative to supply chain considerations
- Create clear workflows for testing sites to direct swabs for in-house lab versus send-out testing
- Create team and process for follow-up on test results, distancing, and disease process counseling via telephone, chat, or phone app
- Establish a quality assurance process for testing site staff, telehealth clinicians, results reviewers, and any other groups involved
- Create ongoing process improvements to address obstacles, questions, and changes 
- Ensure that each step has specific team members and each team has a specific role and defined leadership. This ensures improved flow and efficiency, and allows challenges to be quickly identified and fixed. 

### Limitations

We acknowledge certain limitations to this innovation. One challenge appeared on Monday, March 16, 2020, when the telehealth platform software functionality slowed in processing speed. We quickly identified the cause, a large increase in the number of clinicians seeing patients. This was rectified by Teladoc by scaling the cloud-based resources. We considered this a learning lesson; we needed to better anticipate technology limitations and associated rapid growth in users.

Prior to centralization of testing and registration by Seamless Access, patients were required to call separate telephone numbers for different testing sites. Understandably, this caused confusion for both patients and for clinicians. The addition of Seamless Access and a centralized telephone number to call for registration and testing site information streamlined the workflow. 

There is a cost to the service, which can also be a barrier to employing this model of care delivery, in addition to the requirement for a smartphone, tablet, or computer and internet connection among users that may limit this as an access point to care.

### Conclusion

Telehealth has arrived as a crucial component of testing and treatment during the COVID-19 crisis. In a matter of days, our use of telehealth transformed the way Jefferson Health was providing patient care during a pandemic. Developing a successful and scalable telehealth solution is allowing patients to stay home and preserve hospital resources as much as possible, receive testing for COVID-19 when appropriate, and get directed to additional care when necessary. Additional research will be required to determine the effectiveness of telehealth in reducing exposures for health care workers and the community. However, in this time of uncertainty, patients are seeking evidence-based advice and the benefits of virtual care have become clear to clinicians, patients, and the health care system.

## Abbreviations

APP: advanced practice provider
ED: emergency department
EM: emergency medicine
FM: family medicine
IM: internal medicine
PPE: personal protective equipment
